# Wound Healing after Acellular Dermal Substitute Positioning in Dermato-Oncological Surgery: A Prospective Comparative Study

**DOI:** 10.3390/life13020463

**Published:** 2023-02-07

**Authors:** Alessia Paganelli, Andrea Giovanni Naselli, Laura Bertoni, Elena Rossi, Paola Azzoni, Alessandra Pisciotta, Anna Maria Cesinaro, Luisa Benassi, Shaniko Kaleci, Federico Garbarino, Barbara Ferrari, Chiara Fiorentini, Camilla Reggiani, Cristina Magnoni

**Affiliations:** 1 Dermatologic Surgery Unit, Modena University Hospital, Via del Pozzo 71, 41124 Modena, Italy; 2 PhD Course in Clinical and Experimental Medicine, University of Modena and Reggio Emilia, 41121 Modena, Italy; 3 Department of Surgery, Medicine, Dentistry and Morphological Sciences with Interest in Transplant, Oncology and Regenerative Medicine, 41124 Modena, Italy; 4 Department of Anatomic Pathology, Modena University Hospital, 41124 Modena, Italy

**Keywords:** dermal substitute, dermatologic surgery, skin cancer, wound healing, collagen

## Abstract

Background: MatriDerm and Integra are both widely used collagenic acellular dermal matrices (ADMs) in the surgical setting, with similar characteristics in terms of healing time and clinical indication. The aim of the present study is to compare the two ADMs in terms of clinical and histological results in the setting of dermato-oncological surgery. Methods: Ten consecutive patients with medical indications to undergo surgical excision of skin cancers were treated with a 2-step procedure at our Dermatologic Surgery Unit. Immediately after tumor removal, both ADMs were positioned on the wound bed, one adjacent to the other. Closure through split-thickness skin grafting was performed after approximately 3 weeks. Conventional histology, immunostaining and ELISA assay were performed on cutaneous samples at different timepoints. Results: No significant differences were detected in terms of either final clinical outcomes or in extracellular matrix content of the neoformed dermis. However, Matriderm was observed to induce scar retraction more frequently. In contrast, Integra was shown to carry higher infectious risk and to be more slowly reabsorbed into the wound bed. Sometimes foreign body-like granulomatous reactions were also observed, especially in Integra samples. Conclusions: Even in the presence of subtle differences between the ADMs, comparable global outcomes were demonstrated after dermato-oncological surgery.

## 1. Introduction

Human skin acts as a barrier against external agents and pathogens [1], prevents water loss [2] and is crucial for vitamin D metabolism [3]. Therefore, loss of cutaneous integrity triggers an evolutionary-conserved sequential mechanism of wound healing aimed at restoring skin architecture [4,5].

Wound healing is composed of four different phases (hemostasis, inflammation, proliferation, and remodeling), and requires a complex orchestration of interactions among many different types of cells, including not only keratinocytes and fibroblasts, but also immune cells, endothelial cells, macrophages, and mesenchymal stromal cells (MSCs) [6,7,8].

Impairment in any phase of the wound healing process could lead to chronic ulcer formation and/or aberrant scarring (e.g., excessive retraction, hypertrophic/keloidal scars). The presence of specific risk factors, such as diabetes and peripheral vascular disease, has proven to be associated with impaired wound healing [9]. Such conditions do not only have a direct impact on patient quality of life, but also represent a burden in term of costs for the healthcare system [10].

For these reasons, dermatological research in the regenerative setting is aimed at finding innovative strategies for complete skin regeneration with restoral of physiological skin architecture [11]. However, at present, only up to about 80% of the skin’s original tensile strength is regained in optimal healing conditions [12].

In the dermato-oncological setting, surgical excision of cutaneous neoplasms brings the implicit need for therapeutic interruption of skin integrity, which sometimes poses great challenges in terms of reconstructive and healing strategies.

For small wounds, primary suture immediately after surgical excision represents the gold-standard treatment [13]. Larger skin defects often impose a need for using different techniques for skin reconstruction [14]. One possibility is using advanced dressings for inducing faster secondary-intention healing [15,16] Possible alternatives include covering the wound surface through in vitro expanded epidermal sheets [17], skin grafts or flaps [18,19], and even the use of nanotechnologies [20], and/or stem-cell based therapies [21].

Currently, skin grafts and flaps represent the most commonly used strategies in dermatologic surgery. The use of cutaneous flaps in dermato-oncology is sometimes limited by tumor and subsequent wound size, while the only fundamental requirement for successful grafting is represented by a well-vascularized wound bed. Moreover, grafts also represent the reconstructive strategy of choice when tumor margins are not clearly definable with non-invasive techniques (such as dermoscopy, confocal microscopy, and OCT) and/or Mohs surgery is not feasible. However, grafts alone sometimes provide unsatisfactory functional and aesthetic results, possibly due to both scar contractures and excessive wound depth, with subsequent apparent depression of the grafted area [22].

In recent decades, acellular dermal matrices (ADMs) have widely been employed in this setting with the aim of reducing scarring and replacing the excised dermal compartment [23].

Various bioengineered scaffolding materials have been developed in order to provide a provisional template for skin cell migration and proliferation, therefore promoting wound healing and reducing scar tissue formation [24].

Two collagenic ADMs, Matriderm (MedSkin Solution Dr. Suwelack AG, Billerbeck, Germany) and Integra (Integra LifeSciences, Plainsboro, NJ, USA) are currently commonly used in dermato-oncological reconstructive surgery, and represent the two available options in our center [25,26].

The primary goal of the present study was to evaluate and compare the clinical outcomes of the two different ADMs using the Vancouver Scar Scale (VSS) and assessing the occurrence of ADM-specific adverse events. We also aimed at assessing ADM-induced architectural changes in the extracellular matrix (ECM) at a histopathological level. Detection of specific cell populations (e.g., myo-fibroblasts, endothelial cells, MSCs) and quantification of ECM components were also considered as secondary objectives of our research.

## 2. Materials and Methods

### 2.1. Study Design

An interventional, prospective, comparative clinical pilot study was performed at the Dermatological Surgery Unit of Modena University Hospital. The study was approved by our institutional review board (Protocol n. CE 4342/20), and all study procedures were performed in accordance with Helsinki declaration principles. Written informed consent was obtained from all participants before undergoing any study procedures.

Ten consecutive patients with medical indications to undergo demolitive dermatologic surgery for large skin cancers, not suitable for classical reconstruction with flaps, were included in the present study. Exclusion criteria included: age < 18 years old; inability to give informed consent or to complete the procedures required for study completion; pregnancy or breastfeeding; known allergy to any component of the dermal substitute; lesions located on palms, soles, genitalia, or in the face area; lesions involving bone and/or periosteum; immunodeficiency; heavy smoking (>10 pack/year); uncontrolled diabetes, osteomalacia, thyroid disorders; connective tissue diseases.

### 2.2. Investigational Study Devices

Two ADMs were used, MatriDerm and Integra. Integra (Integra LifeSciences, Plainsboro, NJ, USA) is a device composed of two layers: a synthetic dermis made of a bovine collagen lattice covalently linked to chondroitin-6-sulfate, derived from shark cartilage, and covered with a silastic epidermis (silicone sheet) [27]. Matriderm (MedSkin Solution Dr. Suwelack AG, Billerbeck, Germany) is a highly porous membrane composed of three-dimensionally coupled collagen and elastin. Bovine dermis is used to obtain the collagenic part of the matrix, while elastin is obtained from the bovine nuchal ligament by hydrolysis [28]. Despite Matriderm being often applied underneath a split-thickness skin graft in the setting of one-stage surgery [29,30], we used both ADMs according to a two-step protocol. Trans-epidermal water loss (TEWL) in the Matriderm-treated area was minimized through external dressings with paraffin gauze and external bandages, due to a lack of silicone coverage of this device. In both cases, 2 mm-thick templates were used.

### 2.3. Study Protocol

**Figure 1:**
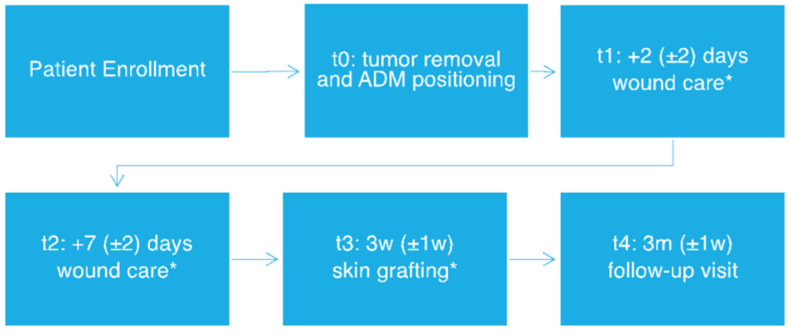
Study workflow. * Tissue sample collection. w: week, m: months.

Patients underwent a two-step surgical procedure with initial dermal substitute positioning and subsequent skin grafting (for protocol flowchart, see Figure 1). Both the ADMs were positioned in the wound bed, with half of the treated area being covered with MatriDerm and the other half with Integra, in order to perform intra-patient comparison, therefore eliminating potential biases due to interindividual variability.

**Figure 2:**
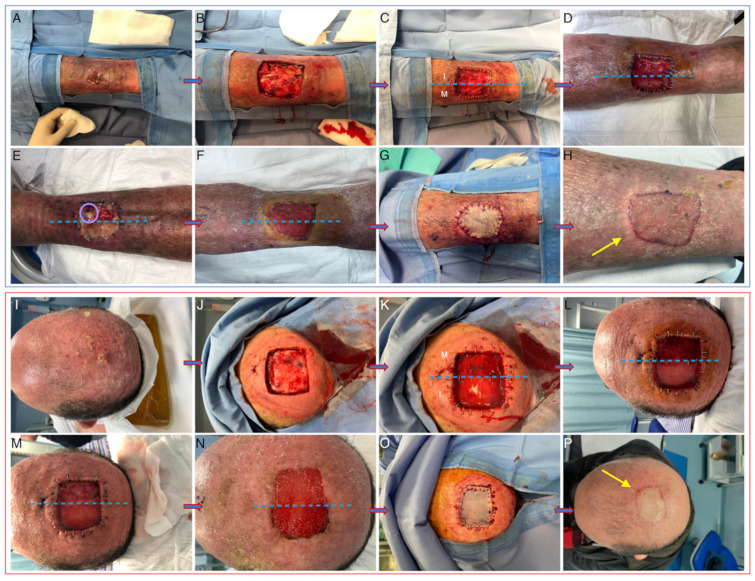
Clinical pictures taken at baseline (**A**,**I**), after tumor removal (**B**,**J**), immediately after ADM positioning (**C**,**K**), at t1 (**D**,**L**), at t2 (**E**,**M**), at t3 before (**F**,**N**) and after (**G**,**O**) skin grafting, and after 3 months (**H**,**P**). Bacterial colonization is evident in panel E for Integra (purulent exudate circled in pink). Yellow arrows indicate slight retraction in Matriderm-treated areas. I: integra, M: Matriderm.

After ADM positioning, all patients included in the study underwent standard wound-care visits at our center twice weekly. Iodopovidone and saline solution were used respectively on Integra and Matriderm and non-adherent dressings were then positioned directly on the ADMs. Microbiological swabs were performed in case of clinical signs of infection. Skin-graft reconstruction (with 0.5–0.6 mm grafts taken either from the thigh or the axillary region) was performed after 3 weeks from the first surgical intervention, after histological confirmation of complete excision with clear margins. External non adherent dressings were applied directly on the graft and changed twice weekly until suture stitch removal. Clinical pictures were collected immediately before and after the first surgical procedure (t0), at the first two post-surgical visits (t1 and t2), at the time of grafting (t3), after 3 months from surgery (t4) (See Figure 2).

ADM samples were also collected at t1 and t2 on days 2 and 7 (±2), respectively, after ADM positioning. Skin biopsies were performed intraoperatively during the second surgical session with a 4-mm punch (t3).

Long-term clinical outcomes were also evaluated 3 months later, during follow-up visits (t4); the Vancouver Scar Scale [31] was used for surgical scar evaluation at t4.

### 2.4. Conventional Histology

ADM and/or skin samples obtained at t1, t2 and t3 were fixed in formalin, paraffin-embedded, and stained with conventional hematoxylin and eosin (HE). At t1 and t2, ADM colonization and the presence of inflammatory infiltrate were evaluated. At t3, several histopathological parameters were considered to investigate cellular and structural characteristics of the neodermis: persistence of dermal substitute, vascularization, granulation phase (early/late), re-epithelization, abundance and type of the inflammatory infiltrate, presence of foreign-body reaction. Histopathological specimens were evaluated and quantified in three random sections of each sample by a panel of three blinded experts. Histological images were obtained using a Nikon Labophot-2 light microscope with a DS-5Mc CCD camera. As for vascularization, immunohistochemical staining for CD31 (BK3528S PECAM-1, Cell Signaling, Danvers, MA, USA) was performed on deparaffinized sections obtained at t3 to confirm histological data on vascularization. Immunohistochemical images were obtained using a Nikon Labophot-2 light microscope with a DS-5Mc CCD camera. Finally, vessels were also selected on acquired images (3 sections for each sample) through manual selection of CD31+ vessels, and the vascularized area was calculated through ImageJ software (version 1.45b) and expressed as percentage area occupied by vessels/total area.

### 2.5. Immunofluorescence

OCT (Tissue-Teck)-embedded culture samples were cryopreserved at −80 °C and used to prepare 4 μm thick sections using a cryotome (LEICA 1720 rotary cryotome, Nussloch, Germany). An immunofluorescent (IF) stain for alpha-SMA (smooth muscle actin) and both single stain and co-staining for CD90 and Stro-1 were performed on these sections. Immunofluorescent stains were repeated on deparaffinized sections, due to poor quality of the neodermis architecture appreciable after freezing and thawing.

After specific site blockage with PBS/BSA, the neodermis sections were incubated with mouse anti-Stro1 and rabbit anti-CD90 antibodies (MAB1038, RD systems; JF-10-09, Invitrogen) for 1 h at room temperature. A signal was visualized with goat polyclonal anti-mouse FITC-conjugated and swine anti-rabbit TRITC-conjugated secondary antibodies (Abcam, Cambridge, UK).

Finally, sections were rinsed, permeabilized with 0.1% Tryton X-100 for 5 min at 4 °C and counterstained with 1μg/mL 4′,6-diamidino-2-phenylindole DAPI (DAPI, Sigma Aldrich, St. Louis, MO, USA) for another 5 min at room temperature. Sections were rinsed and mounted on glass slides before evaluation under a Nikon A1 confocal laser scanning microscope. The confocal serial sections were processed with ImageJ software and image rendering was performed using Adobe Photoshop Software.

### 2.6. ELISA Test

Frozen samples taken at t3 were preserved at −80 °C for 4–8 weeks. Tissue samples were then processed for protein extraction with lysis buffer according to our previously protocol [32]. The ELISA test for type-I collagen and fibronectin was performed according to manufacturer’s instructions (Col 1 kit—Cloud-Clone Corporation, Katy, TX, USA; Human Fibronectin kit ab219046, Abcam, UK). ELISA tests were repeated at two different dilutions and mean values were considered for statistical analyses.

### 2.7. Statistical Analysis

Statistical analysis was performed using STATA^®^ software version 14 (StataCorp. 2015. Stata Statistical Software: Release 14. College Station, TX, USA: StataCorp LP.). Numerical data were expressed as mean, standard deviation, and range. Qualitative data were expressed as frequency and percentage. Chi-square test (Fisher’s exact test) and Student’s *t*-test was used to examine the relation between qualitative variables and continuous variables. A *p*-value < 0.05 was considered significant.

## 3. Results

### 3.1. Baseline Patient Characteristics

Seven of the enrolled patients were men and three were women. Age ranged between 58 and 95 years (mean 84.2). None of our patients were active smokers (See Table 1). Cardiovascular risk factors and heart disease were found to be common comorbidities in our cohort (including type-II diabetes, vasculopathy and arterial hypertension). As for the types of excised lesions, four were basal-cell carcinomas (BCCs), four squamous-cell carcinomas (SCCs) and two atypical fibroxanthomas (AFXs) (Table 1).

### 3.2. Clinical Outcomes

No significant differences between the two substitutes in terms of global VSS were observed. However, more evident wound bed contracture was evident in four cases in areas treated with Matriderm (*p* < 0.05; see Figure 2). One patient experienced a stroke after hospital dismission and required subsequent hospitalization in the ICU, but such an episode was not considered to be related to the use of ADMs. No other major adverse events were recorded during the entire study duration. With regard to minor adverse events, signs of surgical wound infection were detected in five subjects, with Integra being more easily colonized by bacteria compared to Matriderm (*p* = 0.05). All the infections occurred between t1 and t2. Of these, only three cases were considered to be critical colonization, and they completely resolved after partial silicone layer removal and topical antibiotics and/or antiseptics. Microbiological swabs were performed in all cases refractory to local treatment, and results were positive for Pseudomonas aeruginosa and Proteus mirabilis. Two patients required total silicone layer removal and systemic oral antibiotic therapy for complete resolution of the local infection. No significant infectious sequelae were detected during the observation period. In one case only, tissue samples obtained at t3 were not adequate for performing all the punch biopsies required for further laboratory analyses due to severe tissue damage secondary to infection.

### 3.3. Neodermis Architecture and Composition

**Figure 3:**
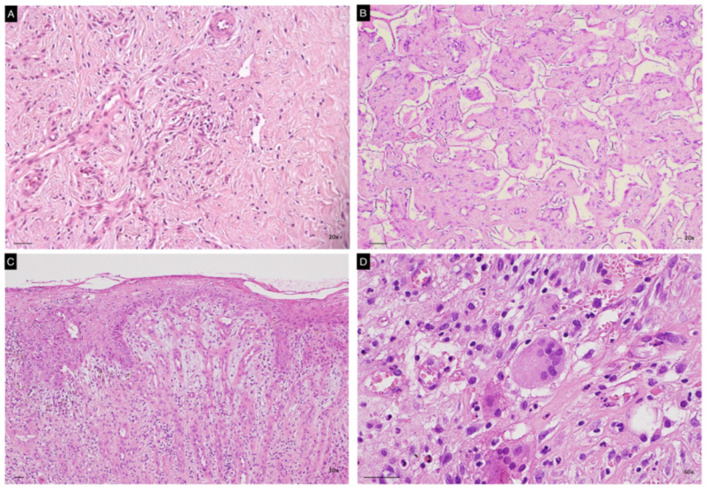
Representative images of histopathological specimens from wound bed at t3 of both Matriderm (**A**,**C**) and Integra (**B**,**D**). Panel B clearly shows persistence of the ADM in the neodermis. Epithelization of the treated area is evident in panel C. A giant multinucleated cell is present in panel D. Scale Bar 50 um.

Histopathological evaluation of cutaneous specimens at t3 led to the detection of significant differences in terms of ADM persistence and quality of the granulation tissue (see Table 2). Matriderm was shown to be re-absorbed more quickly than Integra (*p* < 0.005, see Figure 3, panels A,B). Faster maturation of the granulation tissue was also observed in Matriderm-treated areas (*p* < 0.05), with some of these also being re-epithelized (Figure 3, panel C). No significant differences between the two dermal scaffolds were detected in terms of inflammatory infiltrate, mostly being composed of both neutrophils and lymphocytes, but occasionally also containing eosinophils. Despite not being statistically significant, granulomatous reactions with giant multinucleated cells were more frequently observed within Integra-treated wound beds (Figure 3 panel D).

**Figure 4:**
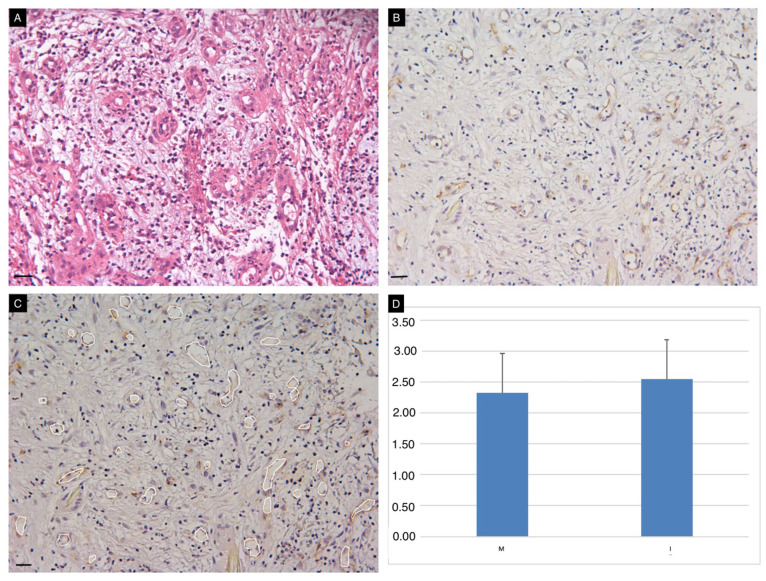
Vascularization of the neodermis. Conventional HE (**A**) and immunohistochemical (**B**) staining for CD31 on tissue sections obtained at t3. Image J Software was used for semi-quantitative assessment of vascularized areas (white circles, panel **C**). Vascularization of the wound bed (expressed as % of the total area) occurred in all cases, with no significant differences between the two ADMs (**D**). Scale Bar 50 um.

Vascularization of the wound bed was detected in all cases. The presence of vessel-like structures in the neodermis observed with classical HE stains was confirmed by CD31 immunostaining (Figure 4, panels A,B). Quantitative assessment of vascularized areas confirmed similar vascularization of the wound bed with the use of the two ADMs (Figure 4, panel C,D).

From a quantitative point of view, no significant differences in terms of collagen and fibronectin content were detected between the two substitutes (see Table 2).

### 3.4. Cellular Colonization of the ADM

**Figure 5:**
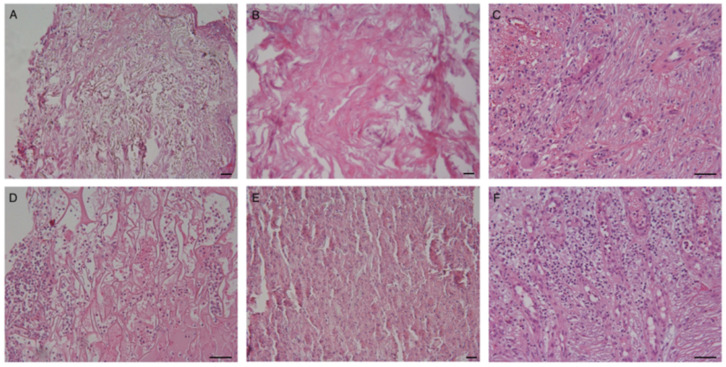
Cellular colonization of the ADM. (**A**–**C**) Integra; (**D**–**F**) Matriderm. Cells are already present in the dermal substitutes after 2 days from ADM positioning. Granulocytes and erythrocytes are the most prevalent cells at t1 and t2 (panels **A**,**B**,**D**,**E**). Fibrinous material is sometimes also evident at t2 (panel **B**). The inflammatory infiltrate is often present in the newly formed dermis. A mixed inflammatory infiltrate is observable at t3 (panels **C**,**F**), with occasional presence of multinucleated cells (panel **C**). Scale Bar 100 um.

**Figure 6:**
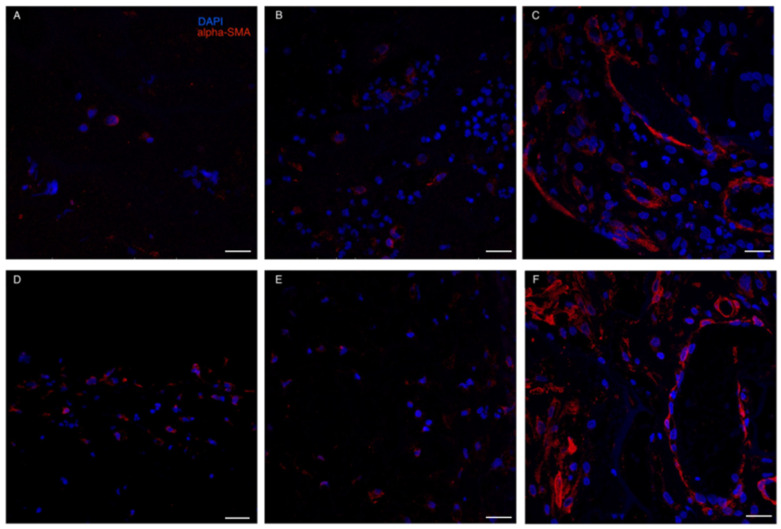
Immunofluorescence staining for α-SMA. (**A**–**C**): Integra; (**D**–**F**): Matriderm. Immunofluorescence analyses confirmed the ongoing rearrangement of cells, including apha-SMA + pericytes at t3 in both ADM (panels **C**,**F**). Weaker positivity was present at t1 (**A**,**D**) and t2 (**B**,**E**), possibly due to the presence of myofibroblasts. Scale Bar 50 um.

Red blood cells and granulocytes were the most prominent infiltrating cells initially found to colonize both ADMs (Figure 5). IF stain for alpha-SMA demonstrated that myo-fibroblasts were already present at t1-t2 in both ADMs. However, a more consistent presence of alpha-SMA positive cells was detected at t3: not only were myofibroblasts present in the neodermis, but dermal vessels also demonstrated a strong positivity for α-SMA, probably due to its expression by capillary pericytes [33] (Figure 6).

**Figure 7:**
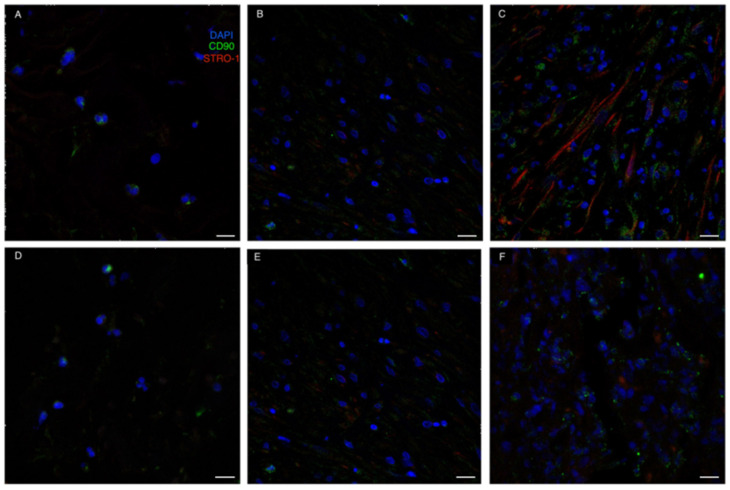
Immunofluorescence staining for MSC-related markers. (**A**–**C**): Integra; (**D**–**F**): Matriderm. CD90 (green) was already expressed by some cells colonizing the ADM at t1. On the contrary, STRO-1 expression was a bit delayed compared to CD90, with STRO-1 positive cells (red) only being present from t2. The recruitment of mesenchymal stromal cells (CD90 and STRO-1 positive cells) in the regenerating areas was evident at t3 (panels **C**,**E**). Scale Bar 20 um.

As for MSC-specific markers, CD90 was already expressed by some cells colonizing the ADM at t1. On the contrary, Stro1 expression was delayed compared to CD90, with Stro1 positive cells only being present from t2. However, MSCs co-expressing both CD90 and Stro1 were only evident in the neodermis at t3 (Figure 7).

## 4. Discussion

Despite being widely used in reconstructive surgery, ADMs lacked standardized randomized controlled trials supporting their efficacy in the dermatological setting [34,35,36,37,38]. More data are currently available on their use in breast reconstruction after mastectomy, where ADMs are becoming routinely employed [39]. However, conflicting data are currently emerging from updated observations of ADM-specific side effects, therefore suggesting careful patient selection for ADM-based reconstructive surgery [40,41].

Recently, Lohmander et al. [42,43] published the results of a milestone study aimed at assessing the differences in breast reconstruction after mastectomy with and without the use of ADMs. The authors found no significant differences between immediate implant-based breast reconstruction and reconstruction with the use of ADMs in terms of reinterventions or surgical complications, health-related quality of life or patient-reported aesthetic outcome. To date, however, it is impossible to draw similar conclusions regarding the use of ADMs for post-oncological surgery skin wound healing.

Various studies have already widely explored the mechanisms of action of dermal substitutes in animal models of wound healing [44,45]. ADMs have already proven not only to provide a collagenic scaffold that increases the dermal thickness, thereby limiting cicatricial depression in healing skin, but also promoting the secretion of endogenous type I and type III collagen in a more physiological manner compared to standard secondary-intention wound healing [44,46].

Only a few comparative studies on ADMs have been published so far on ADM efficacy and potential morphological differences in the neoformed tissue [47,48]. Some of those data specifically focus on Matriderm and Integra, which currently represent two widely used dermal regenerative templates. A study by Joerg Schneider and collaborators [45] aimed to assess differences between Matriderm- and Integra-induced skin regeneration in a rat model of wound healing. The authors found no major differences in engraftment rates, quality of neodermis, or vascularization. Those data were confirmed by Bottcher-Haberzeth et al. in 2012, who did not find statistically significant differences between the two ADMs in terms of neodermis thickness [49].

Most of the in-vivo clinical studies in humans available in the dermatological setting have been performed on surgical reconstruction due to burn wounds and in various surgical sites [50].

In 2020 Philips and coauthors [51] performed a retrospective study aimed at comparing the use of two-stage Integra and single-stage Matriderm at their burn referral center. Comparable grafting rates were observed in both groups. Infections were more common in the Integra group, in line with our data. No significant differences were detected in hematoma development, hypertrophic scarring, or need for secondary surgery. The authors concluded that Integra could be recommended for larger burns with limited donor sites, while Matriderm was preferable for smaller burns in cosmetically sensitive areas.

A second comparative study was conducted in 2020 by Vana et al. [50], who prospectively analyzed clinical and histopathological outcomes in patients treated with two mm-thick Matriderm or Integra, followed by thin skin autografts (two-step procedure) for burn scars healed with sequelae (e.g., with limited mobility and/or bad aesthetic results). Negative pressure therapy was also applied after surgery. Improvement in mobility and skin quality were demonstrated along with graft contraction, in all patients. No intra- or post-operative adverse events were recorded. The authors found that Integra had lower retraction rates and better skin quality compared to Matriderm. In line with our observations, the authors confirmed the tendency of Integra to persist for longer in the newly formed dermis. In contrast with such publications [50], we did not observe significant differences in terms of VSS total scores. However, VSS remains an operator-dependent parameter, thus potentially leading to evaluation bias. Moreover, we confirmed a slight tendency to wound contraction for the Matriderm-treated portion of the wound bed, in line with the Brazilian study mentioned above [50]. Current research suggests that skin pliability and reduced wound contracture are more easily achieved through the use of cross-linked scaffolds [52]. 

Recently, a prospective randomized controlled clinical trial, including patients with burn contracture treated using autologous skin grafts and ADMs, was performed by Corrêa and collaborators [53]. Interestingly, the control group (patients treated with skin graft only, without previous ADM positioning) displayed lower rates of wound contraction. No significant differences were detected between Integra^®^ and Matriderm^®^.

As for the dermato-oncological setting, an Italian group already assessed the use of the two ADMs after craniofacial surgery, both with single- and two-step surgery [54]. The authors detected better performances by Matriderm in terms of skin thickness when used in two-step surgery, while Integra was shown to achieve better results for engraftment and clinical outcomes when applied directly to the bone. We therefore decided not to enroll patients where the periosteum could not be preserved due to the established superiority of Integra in such cases. The main differences between our study and the one by Torresini and Gareffa reside in the retrospective nature of their observations; the possible selection biases due to interindividual variability in wound healing; the intrinsic heterogeneity of the surgical procedures (both one and two-step interventions were considered); the lack of histological evaluation. On the other hand, the limited number of patients enrolled in our pilot study represents a possible limitation in extending our findings to larger-scale casuistries.

Compared to the other available studies, our work has one major strong point: Matriderm and Integra Bilayer are both positioned in a single surgical wound bed, with each single patient enrolled acting both as a case and as a control, thus eliminating any eventual confounding selection bias. Notwithstanding, the main limit of our study resides in the relatively small number of patients enrolled. Some minor concerns regarding our data could also arise due to the use of Matriderm in a two-step procedure. In fact, Matriderm is generally used in one-stage surgery with concomitant skin grafting, with a 3-week interval between ADM positioning and grafting possibly being associated with wound bed retraction.

In line with previously published data [51], we observed higher rates of infection in patients treated with Integra. A possible explanation could reside in the occlusion provided by the silicon membrane present in the Integra device, which could favor bacterial proliferation. Several studies postulated specific skin cancer types to be associated with surgical-site infections [55,56], the most recent ones indicating SCC as possibly carrying the higher risk. Our data do not allow us to draw similar conclusions with significant statical strength: of the five cases observed, three were detected in SCCs and two in BCCs. However, no cases of infection were detected among cutaneous neoplasms other than NMSCs, such as AFX.

Quantitative analysis though ELISA testing confirmed the lack of significant differences between the two devices in terms of ECM production in the neo-dermis. Histopathologically, the newly formed dermis was well vascularized in both Matriderm and Integra, without relevant differences. Moreover, Matriderm was found to more often be reabsorbed in the first weeks after ADM positioning compared to Integra. Probably collagen crosslinking in such ADM could give a partial reason for the observed variability in reabsorption rates. Furthermore, immunofluorescence analyses confirmed the ongoing rearrangement of cells, including apha-SMA + pericytes, involved in neovascularization processes occurring in wound healing. Also, the recruitment of mesenchymal stromal cells in the regenerating areas was evident, as shown by the immunolabeling against CD90 and STRO-1, demonstrating shared features between the two ADMs.

In conclusion, no significative differences have been found between Matriderm and Integra in our prospective comparative study, both in terms of clinical efficacy and histopathological findings. However, more data are needed to extend our results to a larger casuistry, thereby possibly guiding daily clinical practice. 

## Figures and Tables

**Table 1:** Patient and wound baseline characteristics.

Sex	Age	CV Comorbidities	Histological Dx	Body Site
M	95	0	SCC	scalp
F	82	1	SCC	LL
F	87	0	BCC	LL
M	90	1	SCC	scalp
F	90	1	BCC	scalp
M	81	1	SCC	scalp
M	84	0	BCC	LL
M	89	1	BCC	LL
M	58	1	AFX	scalp
M	86	1	AFX	scalp

CV: cardiovascular; Dx: diagnosis; M: male; F: female; SCC: squamous cell carcinoma; BCC: basal cell carcinoma; AFX: atypical fibroxanthoma; LL: lower limbs.

**Table 2:** Clinical parameters, histopathological parameters and tissue quantitative assessment. Values are expressed as percentages (%) and/or mean ± SD (range).

		I		M		Total		
		N	%	N	%	*n*	%	*p*-Value
Infection	N	5	50	9	90	14	70	0.051
	Y	5	50	1	10	6	30	
Fibronectin (ug/100 uL)	mean ± SD (range)	0.7 ± 0.3 (0.4–1.2)		0.8 ± 0.4 (0.4–1.8)		07 ± 0.4 (0.4–1.8)		0.716
Collagen (ug/100 uL)	mean ± SD (range)	2.0 ± 0.4 (1.3–2.8)		2.1 ± 0.4 (1.4–2.8)		2.0 ± 0.4 (1.3–2.8)		0.737
ADM persistence	N	2	20	7	80	8	40	0.004
	Y	8	80	2	20	10	50	
Granulation tissue	immature	8	80	4	40	12	60	0.046
	mature	1	10	5	50	6	30	
Epithelization	absent	10	100	8	80	18	90	0.136
	present	0	0	2	20	2	10	
Inflammation	mild	5	50	6	60	11	55	0.639
	severe	4	40	3	30	7	35	
Granulomatous reaction	N	6	60	8	80	14	70	0.257
	Y	3	30	1	10	4	20	
Eosinophils	N	8	80	7	70	15	75	0.527
	Y	1	10	2	20	3	15	
Vascularized area (%)	Mean ± SD (range)	2.5 ± 1.3 (0.0–4.1)		2.2 ± 0.6 (0.0–4.1)		2.3 ± 1.30 (0.0–4.1)		0.584
VSS	Mean ± SD (range)	5.7 ± 1.9 (2–8)		6.1 ± 1.6 (3–8)		5.9 ± 1.7 (2–8)		0.580

I: Integra; M: Matriderm; *n*: number; N: No; Y: Yes; ADM: Acellular Dermal Matrix; VSS: Vancouver Scar Scale.

## Data Availability

Data are available from the authors upon reasonable request.
